# Knowledge, attitudes, and practices of emergency nurses regarding sepsis nutritional management: a multicenter cross-sectional study

**DOI:** 10.3389/fnut.2026.1933045

**Published:** 2026-10-08

**Authors:** Lin Xiao, Weijia Pei, Huijun Zhu

**Affiliations:** Department of Emergency, Affiliated Hospital of Yangzhou University, Yangzhou, Jiangsu, China

**Keywords:** attitudes, knowledge, nurses, nutritional management, practice

## Abstract

**Background:**

Nutritional management is an important component of supportive care for patients with sepsis, but its implementation in emergency settings may be challenging alongside other time-critical priorities. This study assessed emergency department (ED) nurses’ knowledge, attitudes, and practices (KAP) regarding nutritional management in patients with sepsis.

**Methods:**

This multicenter cross-sectional study was conducted among ED nurses from hospitals in Jiangsu Province, China, between January and May 2026. A structured Chinese questionnaire with satisfactory internal consistency was used to assess KAP, with KAP levels descriptively classified using modified Bloom’s cut-off criteria. Multivariable linear regression was performed to identify factors associated with KAP scores, and structural equation modeling (SEM) was used to further evaluate the direct and indirect relationships among KAP domains.

**Results:**

A total of 630 valid questionnaires were analyzed, including 558 female nurses (88.6%). The median knowledge, attitude, and practice scores were 9.00 (9.00, 15.00), 40.50 (36.00, 45.00), and 29.00 (22.25, 36.00), respectively, out of possible ranges of 0–18, 9–45, and 9–45. In multivariable analyses, nurses without recent training had significantly lower knowledge scores (β = −4.676, 95% CI: −5.386 to −3.965, *p* < 0.001) and practice scores (β = −3.014, 95% CI: −4.296 to −1.732, *p* < 0.001). Higher knowledge scores were independently associated with more positive attitudes (β = 0.419, 95% CI: 0.337 to 0.501, *p* < 0.001) and better practices (β = 0.860, 95% CI: 0.723 to 0.997, *p* < 0.001), while higher attitude scores were also independently associated with better practices (β = 0.415, 95% CI: 0.294 to 0.537, *p* < 0.001). SEM demonstrated that knowledge had significant direct effects on attitude (β = 0.470, 95% CI: 0.404–0.536, *p* < 0.001) and practice (β = 0.512, 95% CI: 0.432–0.589, *p* < 0.001), and also had an indirect effect on practice through attitude (β = 0.100, 95% CI: 0.061–0.140, *p* < 0.001).

**Conclusion:**

ED nurses showed insufficient knowledge, positive attitudes and moderate practice toward nutritional management in patients with sepsis. The observed associations of knowledge with both attitude and practice support the potential value of targeted, practice-oriented training, together with standardized documentation and workflow-based support.

## Introduction

Sepsis, defined as life-threatening organ dysfunction caused by a dysregulated host response to infection, remains one of the most pressing public health challenges worldwide (1). A Global Burden of Disease analysis estimated 48.9 million incident cases of sepsis and 11.0 million sepsis-related deaths worldwide in 2017, accounting for nearly one fifth of all global deaths (2). The World Health Organization has also emphasized that sepsis is one of the most frequent causes of death worldwide and requires early recognition and coordinated management (3). Although antimicrobial therapy, hemodynamic resuscitation, source control, and organ support remain the cornerstone of sepsis care (4), nutritional status is also clinically relevant, as inflammation-driven catabolism and ICU-acquired muscle wasting are common during critical illness, and poor nutritional indices have been associated with increased 30-day mortality among patients with sepsis (5, 6).

Patients with sepsis are particularly vulnerable to nutritional deterioration because systemic inflammation, hypermetabolism, insulin resistance, and protein catabolism may rapidly accelerate muscle loss and immune dysfunction (7, 8). In a prospective observational study of critically ill septic patients, 58.2% were classified as being at high nutritional risk according to the Nutrition Risk in Critically Ill (NUTRIC) score, underscoring the high burden of nutritional vulnerability in this population (8). International guidance recommends that nutritional therapy in critically ill patients should be based on nutritional risk assessment, gastrointestinal function, hemodynamic stability, energy and protein targets, and careful monitoring of feeding tolerance (5, 9). The 2021 Surviving Sepsis Campaign guidelines also suggest early enteral nutrition within 72 h in adults with sepsis or septic shock who can be fed enterally (4). However, nutritional support in sepsis involves complex, individualized decision-making rather than merely the early initiation of feeding. Evidence from trials and reviews indicates that feeding route, dose, timing, shock severity, vasopressor use, aspiration risk, and gastrointestinal intolerance must be balanced to avoid preventable complications (10–12). These requirements make bedside assessment and nursing surveillance essential to safe nutritional care.

Knowledge, attitude, and practice (KAP) surveys help identify gaps between clinical awareness, perceived importance, and actual implementation, thereby informing targeted training and quality improvement (13, 14). This approach is closely relevant to sepsis nutritional care because guideline-based management depends on whether frontline staff understand and consistently apply key recommendations. In the emergency department (ED), nurses are often the first professionals to assess patients with suspected infection, monitor clinical deterioration, initiate screening pathways, and support time-critical interventions. ED nurses play a practical role in early nutritional risk identification, communication with physicians and dietitians, preparation for enteral feeding, prevention of aspiration, glucose monitoring, documentation of feeding interruption, and patient or family education. Therefore, assessing ED nurses’ KAP regarding nutritional management in patients with sepsis is necessary to identify modifiable barriers in emergency care. However, existing KAP studies among emergency nurses have mainly focused on sepsis management (15, 16), rather than specifically addressing nutritional management. Evidence specifically addressing ED nurses’ KAP regarding nutritional management in patients with sepsis remains scarce.

Therefore, this study aims to assess the knowledge, attitudes, and practices of ED nurses regarding nutritional management in patients with sepsis.

## Methods

### Study design and participants

This multi-center cross-sectional study was conducted among nurses in ED from hospitals in Jiangsu Province, China, between January and May 2026. Ethical approval was obtained from the Ethics Committee of Affiliated Hospital of Yangzhou University. All participants were informed of the study purpose, voluntary nature of participation, anonymity, and confidentiality before completing the questionnaire, and informed consent was obtained electronically before data collection. Eligible participants were ED nurses who: (1) held a valid nursing practice qualification; (2) had over 1 year of clinical nursing experience in the ED; and (3) voluntarily agreed to participate in the survey. Nursing students, interns, trainee nurses, non-clinical personnel, and respondents who did not provide informed consent were excluded.

### Questionnaire

The questionnaire was developed based on current guidelines and expert recommendations specifically related to nutritional management in patients with sepsis, including the *ESPEN practical and partially revised guideline: Clinical nutrition in the intensive care unit* (9) and the *Guidelines for medical nutritional therapy in adult sepsis patients (2025 edition)* (17). After the initial draft was developed, expert consultation was conducted with three clinical nursing experts, each with more than 15 years of experience in emergency nursing, and one nutrition expert. The experts evaluated the relevance, clarity, professional accuracy, and clinical applicability of the questionnaire items, and the questionnaire was revised accordingly. Formal quantitative content validity indices, such as the item-level content validity index (I-CVI) and scale-level content validity index (S-CVI), were not collected during questionnaire development.

A pilot survey involving 39 participants was subsequently conducted to confirm the reliability of the instrument. The Cronbach’s α coefficients were 0.974 for the knowledge dimension, 0.957 for the attitude dimension, 0.968 for the practice dimension, and 0.968 for the overall KAP scale, indicating good internal consistency. During the pilot survey, participants were also asked to provide feedback on the clarity and comprehensibility of the questionnaire items. No difficulties in understanding the wording or content of the items were reported, thereby supporting the face validity of the questionnaire. In the final analytic sample of 630 participants, Cronbach’s α coefficients were 0.967 for the knowledge dimension, 0.935 for the attitude dimension, 0.961 for the practice dimension, and 0.956 for the overall KAP scale, indicating excellent internal consistency of the Chinese version of the questionnaire. Construct validity of the Chinese questionnaire was evaluated using confirmatory factor analysis (CFA), specifying knowledge, attitude, and practice as three latent constructs measured by their respective nine items. The three-factor model demonstrated acceptable fit (Supplementary Table S1). Standardized factor loadings ranged from 0.721 to 0.937 for knowledge, 0.515 to 0.933 for attitude, and 0.784 to 0.953 for practice, supporting the construct validity of the three-domain structure (Supplementary Table S2).

After revision, the final questionnaire was administered in Chinese and consisted of four sections: demographic characteristics, knowledge, attitudes, and practices. Demographic characteristics included sex, age, educational level, work experience, professional title, hospital type, hospital level, and training experience related to critical care nutrition management or enteral nutrition during the past year. Training experience in the past year was defined as self-reported participation during the preceding 12 months in any education or training related to critical care nutritional management or enteral nutrition.

The knowledge dimension included 9 items, and each item was scored as 2 points for “very familiar,” 1 point for “heard of it,” and 0 points for “do not know,” yielding a total knowledge score ranging from 0 to 18. The attitude dimension consisted of 9 items, and each item was rated on a five-point Likert scale from 5 (“strongly agree”) to 1 (“strongly disagree”), with a total attitude score ranging from 9 to 45. The practice dimension also comprised 9 five-point Likert scale items, each rated from 5 (“always”) to 1 (“never”), resulting a total practice score between 9 and 45. The final Chinese-language questionnaire was used for data collection. Reliability and construct-validity analyses reported in this study therefore apply specifically to this Chinese version and should not be assumed to generalize to translated or culturally adapted versions without further validation. Consistent with Bloom’s cut-off criteria commonly used in KAP studies (18, 19), scores for each dimension were classified according to the percentage of the maximum attainable score: <60% was defined as poor, 60%–79% as moderate, and ≥80% as good. These categories were used to facilitate descriptive interpretation rather than as clinically validated diagnostic thresholds.

### Questionnaire distribution and quality control

A convenience sampling method was used. The electronic questionnaire was generated using the Wenjuanxing platform,^1^ through which a QR code and survey link were created for online distribution. Participants completed the questionnaire by scanning the QR code or accessing the link using their mobile phones. At the seven initially contacted hospitals, one designated contact researcher was responsible for coordinating questionnaire distribution. The contact researcher introduced the study purpose and questionnaire requirements to eligible nurses at their hospital and invited them to complete the survey voluntarily. In addition, some questionnaires were distributed during academic conferences attended by ED nurses in Jiangsu Province. To minimize collection of potentially identifying institutional information, the questionnaire did not record individual hospital names or a separate recruitment-source identifier. Therefore, the number of respondents contributed by each hospital and the proportion recruited through conference distribution could not be retrospectively determined. Before accessing the formal questionnaire, participants were required to read the informed consent statement and confirm their willingness to participate. To reduce missing data, all questionnaire items were set as mandatory before submission. To improve data quality, questionnaires were screened according to predefined criteria. To ensure data quality, predefined exclusion criteria were applied before statistical analysis. Responses were regarded as invalid and excluded if they met any of the following criteria: (1) incorrect response to the attention-check item; (2) completion time of less than 90 s, which was considered insufficient for careful reading and completion of the questionnaire; (3) obviously implausible demographic information, such as an age of 180 years; or (4) highly repetitive response patterns, such as selecting the same option for all questionnaire items, suggesting insufficient engagement or potential response bias.

### Sample size

The minimum sample size was estimated according to the commonly used rule for questionnaire-based studies, which recommends a sample size of at least 10 times the number of questionnaire items (20). The formal KAP questionnaire included 27 scoring items, including 9 knowledge items, 9 attitude items, and 9 practice items. After allowing for a potential invalid response rate of 20%, the minimum target sample size was increased to 338 participants.

### Statistical analysis

Statistical analyses were performed using R software version 4.4.3 and AMOS version 24.0 (IBM, Armonk, NY, USA). Continuous variables were assessed for normality using the Shapiro–Wilk test. As the KAP scores deviated from normal distribution (Shapiro–Wilk W = 0.901, 0.866, and 0.971, respectively; all *p* < 0.001), they were summarized as medians with interquartile ranges [M (Q1, Q3)]. Between-group comparisons of KAP scores were therefore performed using the Mann–Whitney U test for two-group variables and the Kruskal–Wallis test for variables with more than two groups. Categorical variables were presented as frequencies and percentages. Spearman correlation analysis was used to evaluate correlations among KAP scores. Univariable and multivariable linear regression analyses were conducted to identify factors associated with KAP scores. Variables with statistical significance in univariable analyses and variables considered clinically relevant were included in multivariable models. Structural equation modeling (SEM) was performed to examine the direct and indirect relationships among KAP domains. Standardized path coefficients were estimated, and bias-corrected bootstrap confidence intervals were calculated using 5,000 resamples. Model fit was evaluated using the chi-square/degrees of freedom ratio (CMIN/DF), root mean square residual (RMR), incremental fit index (IFI), Tucker–Lewis index (TLI), comparative fit index (CFI), and root mean square error of approximation (RMSEA). A two-sided *p* value < 0.05 was considered statistically significant.

## Results

### Basic characteristics of the participants

A total of 724 questionnaires were initially collected. During data screening, 94 responses were excluded based on predefined quality-control criteria: 16 were completed in less than 90 s, 59 failed the attention-check item, 8 contained implausible age values, and 11 showed repetitive response patterns. Consequently, 630 valid questionnaires were retained for the final analysis, yielding a valid rate of 87.0%. The included participants had a mean age of 37.9 ± 7.9 years, 558 (88.6%) were female, and the largest proportion had 11–20 years of work experience (238, 37.8%). In detail, most participants had a junior college education or below (427, 67.8%). Regarding professional characteristics, the majority of participants had 11–20 years of work experience (238, 37.8%) and held a junior professional title or below (458, 72.7%). In addition, 386 (61.3%) participants had not received training related to critical care nutrition management or enteral nutrition in the past year.

The median knowledge, attitude, and practice scores were 9.00 (9.00, 15.00), 40.50 (36.00, 45.00), and 29.00 (22.25, 36.00), respectively, out of possible ranges of 0–18, 9–45, and 9–45. The median knowledge, attitude, and practice scores corresponded to 50.0% (9/18), 90.0% (40.5/45), and 64.4% (29/45) of their respective maximum attainable scores and were therefore descriptively classified as poor knowledge, good attitudes, and moderate practice according to the modified Bloom’s criteria. Significant differences in knowledge scores were observed by sex, age, work experience, professional title, and training experience in the past year (all *p* < 0.05). Attitude scores differed significantly by age, work experience, professional title, and training experience (all *p* < 0.05). Practice scores differed significantly by age, professional title, and training experience (all *p* < 0.05) (Table 1).

**Table 1:** Basic characteristics and KAP scores of included nurses.

Variable	*N* (%)	Knowledge M (Q1, Q3)	*p*	Attitude M (Q1, Q3)	*p*	Practice M (Q1, Q3)	*p*
Total	630 (100.0%)	9.00 (9.00, 15.00)		40.50 (36.00, 45.00)		29.00 (22.25, 36.00)	
Sex			0.012		0.680		0.829
Male	72 (11.4%)	10.50 (9.00, 18.00)		41.50 (35.75, 45.00)		29.00 (22.00, 37.00)	
Female	558 (88.6%)	9.00 (9.00, 15.00)		40.00 (36.00, 45.00)		29.50 (23.00, 36.00)	
Age, years			<0.001		0.011		0.028
≤34	242 (38.4%)	9.00 (8.00, 12.00)		38.00 (36.00, 45.00)		28.00 (21.25, 36.00)	
35–39	140 (22.2%)	9.00 (9.00, 15.25)		41.00 (36.00, 45.00)		29.00 (24.00, 36.00)	
≥40	248 (39.4%)	10.00 (9.00, 17.00)		42.00 (36.00, 45.00)		32.00 (25.00, 37.00)	
Educational level			0.087		0.977		0.900
Junior college or below	427 (67.8%)	9.00 (9.00, 15.00)		40.00 (36.00, 45.00)		29.00 (24.00, 36.00)	
Bachelor’s degree or above	203 (32.2%)	9.00 (9.00, 16.50)		41.00 (36.00, 45.00)		30.00 (22.00, 36.00)	
Work experience, years			<0.001		0.004		0.078
≤5	97 (15.4%)	9.00 (8.00, 11.00)		36.00 (35.00, 43.00)		27.00 (21.00, 35.00)	
6–10	154 (24.4%)	9.00 (8.00, 14.00)		39.00 (36.00, 45.00)		28.00 (24.00, 36.00)	
11–20	238 (37.8%)	9.00 (9.00, 16.00)		42.00 (36.00, 45.00)		30.00 (24.00, 36.00)	
≥21	141 (22.4%)	11.00 (9.00, 17.00)		42.00 (36.00, 45.00)		32.00 (25.00, 38.00)	
Professional title			<0.001		0.006		<0.001
Junior title or below	458 (72.7%)	9.00 (9.00, 13.75)		40.00 (36.00, 45.00)		28.00 (22.00, 36.00)	
Intermediate title or above	172 (27.3%)	12.00 (9.00, 17.00)		42.00 (36.00, 45.00)		33.00 (26.00, 38.00)	
Hospital type			0.230		0.988		0.131
Private hospital	24 (3.8%)	10.50 (9.00, 18.00)		40.00 (36.00, 45.00)		34.00 (28.50, 36.50)	
Public hospital	606 (96.2%)	9.00 (9.00, 15.00)		40.50 (36.00, 45.00)		29.00 (22.00, 36.00)	
Hospital level			0.297		0.101		0.766
Non-tertiary hospital	123 (19.5%)	9.00 (8.50, 14.00)		37.00 (36.00, 45.00)		30.00 (24.50, 36.00)	
Tertiary hospital	507 (80.5%)	9.00 (9.00, 16.00)		42.00 (36.00, 45.00)		29.00 (22.00, 36.00)	
Training experience in the past year			<0.001		<0.001		<0.001
No	386 (61.3%)	9.00 (7.00, 10.00)		38.00 (36.00, 45.00)		27.00 (20.00, 32.75)	
Yes	244 (38.7%)	15.00 (9.00, 18.00)		44.00 (36.00, 45.00)		36.00 (28.75, 40.00)	

Data are presented as *n* (%) for categorical variables and median (Q1, Q3) for knowledge, attitude, and practice scores. Between-group comparisons were performed using the Mann–Whitney U test for two-group variables and the Kruskal–Wallis test for variables with more than two groups. KAP, knowledge, attitude, and practice; Q1, first quartile; Q3, third quartile.

### Item-level distribution of knowledge, attitude, and practice responses

For the knowledge dimension, the item with the highest proportion of “do not know” responses was K3, concerning the use of NRS 2002, NUTRIC, or mNUTRIC for nutritional risk assessment in patients with sepsis, with 101 nurses (16.0%) selecting “do not know.” Other relatively weak knowledge areas included avoidance of early overfeeding (K5: 98, 15.6%), early enteral nutrition under relatively stable hemodynamic conditions and without feeding contraindications (K4: 84, 13.3%), and aspiration risk factors such as impaired consciousness, mechanical ventilation, supine position, reflux, and vomiting (K7: 80, 12.7%) (Table 2).

**Table 2:** Distribution of knowledge dimension responses.

Knowledge items, *n* (%)	Very familiar	Heard of it	Do not know
1. Patients with sepsis are at high risk of malnutrition.	202 (32.1%)	365 (57.9%)	63 (10.0%)
2. Nutritional risk screening should be performed as early as possible in patients with sepsis.	213 (33.8%)	353 (56.0%)	64 (10.2%)
3. Tools such as NRS 2002, NUTRIC, or mNUTRIC can be used for nutritional risk assessment in patients with sepsis.	140 (22.2%)	389 (61.7%)	101 (16.0%)
4. When hemodynamics are relatively stable and there are no contraindications to feeding, early enteral nutrition may be considered for patients with sepsis or septic shock.	187 (29.7%)	359 (57.0%)	84 (13.3%)
5. Early nutritional therapy should not reach full feeding volume at the very beginning, and early overfeeding should be avoided.	149 (23.7%)	383 (60.8%)	98 (15.6%)
6. The infusion method of enteral nutrition should be selected according to the patient’s condition, gastrointestinal tolerance, aspiration risk, and medical orders.	213 (33.8%)	353 (56.0%)	64 (10.2%)
7. Conditions such as impaired consciousness, mechanical ventilation, supine position, reflux, and vomiting may increase the risk of aspiration.	205 (32.5%)	345 (54.8%)	80 (12.7%)
8. When administering enteral nutrition, the name of the nutritional formula, dose/infusion rate, tube position, and medical orders should be checked.	198 (31.4%)	357 (56.7%)	75 (11.9%)
9. During enteral nutrition, signs of feeding intolerance such as abdominal distension, diarrhea, vomiting, gastric retention, reflux, and aspiration should be monitored.	210 (33.3%)	344 (54.6%)	76 (12.1%)

Data are presented as *n* (%).

For most attitude items, more than 90% of participants selected “strongly agree” or “agree.” The highest level of agreement was observed for the item stating that nutritional management is an important component of comprehensive treatment for sepsis, with 355 nurses (56.3%) strongly agreeing and 254 (40.3%) agreeing. Similarly, 358 nurses (56.8%) strongly agreed and 245 (38.9%) agreed that ED nurses need to master nutritional management knowledge for patients with sepsis. However, some hesitation was observed for items related to actively identifying feeding contraindications or aspiration risk and including nutritional management in nursing quality evaluation. For the item stating that nurses should actively pay attention to feeding contraindications or aspiration risk, 68 nurses (10.8%) disagreed and 3 (0.5%) strongly disagreed. For the item stating that nutritional management should be included in nursing quality evaluation for sepsis care, 35 nurses (5.6%) disagreed and 1 (0.2%) strongly disagreed (Table 3).

**Table 3:** Distribution of attitude dimension responses.

Attitude items, *n* (%)	Strongly agree	Agree	Neutral	Disagree	Strongly disagree
1. I believe that nutritional management is an important component of comprehensive treatment for sepsis.	355 (56.3%)	254 (40.3%)	21 (3.3%)	0 (0.0%)	0 (0.0%)
2. I believe that emergency department nurses need to master knowledge related to nutritional management for patients with sepsis.	358 (56.8%)	245 (38.9%)	27 (4.3%)	0 (0.0%)	0 (0.0%)
3. I agree that early identification of nutritional risk by nurses can help promote the subsequent implementation of nutritional therapy.	353 (56.0%)	250 (39.7%)	27 (4.3%)	0 (0.0%)	0 (0.0%)
4. I believe that nurses should actively pay attention to whether patients with sepsis have contraindications to feeding or risk of aspiration.	257 (40.8%)	224 (35.6%)	78 (12.4%)	68 (10.8%)	3 (0.5%)
5. I believe that nurses should actively monitor and promptly report signs of nutritional intolerance to physicians.	320 (50.8%)	262 (41.6%)	47 (7.5%)	1 (0.2%)	0 (0.0%)
6. I believe that emergency departments should establish nursing protocols for enteral nutrition in patients with sepsis.	364 (57.8%)	235 (37.3%)	30 (4.8%)	1 (0.2%)	0 (0.0%)
7. I believe that nutritional management requires multidisciplinary collaboration among emergency physicians, nurses, ICU teams, dietitians, and other professionals.	358 (56.8%)	241 (38.3%)	31 (4.9%)	0 (0.0%)	0 (0.0%)
8. I am willing to regularly receive continuing education related to nutritional management.	352 (55.9%)	243 (38.6%)	34 (5.4%)	1 (0.2%)	0 (0.0%)
9. I believe that nutritional management should be included as part of the nursing quality evaluation for sepsis care.	251 (39.8%)	241 (38.3%)	102 (16.2%)	35 (5.6%)	1 (0.2%)

Data are presented as *n* (%).

The weakest practice item was recording nutrition-related information such as body weight, appetite, and food intake, for which 250 nurses (39.7%) reported “rarely” or “never.” Another relatively weak behavior was recording the reasons for interruption of enteral nutrition, such as examinations, transfer, surgery, tube-related problems, gastrointestinal intolerance, or changes in condition, with 206 nurses (32.7%) reporting “rarely” or “never.” In addition, 204 nurses (32.4%) rarely or never assisted in completing nutritional risk screening or nutrition-related assessment according to departmental requirements (Table 4).

**Table 4:** Distribution of practice dimension responses.

Practice items, *n* (%)	Always	Often	Sometimes	Rarely	Never
1. I record nutrition-related information such as the patient’s body weight, appetite, and food intake.	79 (12.5%)	133 (21.1%)	168 (26.7%)	147 (23.3%)	103 (16.3%)
2. I assist in completing nutritional risk screening or nutrition-related assessment according to departmental requirements.	101 (16.0%)	158 (25.1%)	167 (26.5%)	140 (22.2%)	64 (10.2%)
3. Before administering enteral nutrition, I check the medical orders, nutritional formula, infusion rate, tube position, and patient identity.	119 (18.9%)	193 (30.6%)	162 (25.7%)	111 (17.6%)	45 (7.1%)
4. I pay attention to whether the patient has signs of feeding intolerance, such as vomiting, abdominal distension, diarrhea, reflux, and gastric retention.	115 (18.3%)	166 (26.3%)	153 (24.3%)	133 (21.1%)	63 (10.0%)
5. I record the reasons for interruption of enteral nutrition, such as examinations, transfer, surgery, tube-related problems, gastrointestinal intolerance, or changes in the patient’s condition.	79 (12.5%)	145 (23.0%)	200 (31.7%)	139 (22.1%)	67 (10.6%)
6. I observe whether the patient has risk factors for aspiration, such as impaired consciousness, supine position, reflux, and mechanical ventilation.	110 (17.5%)	188 (29.8%)	171 (27.1%)	111 (17.6%)	50 (7.9%)
7. When administering enteral nutrition, I perform position management as required, such as elevating the head of the bed.	113 (17.9%)	184 (29.2%)	181 (28.7%)	103 (16.3%)	49 (7.8%)
8. When the patient develops feeding intolerance, suspected aspiration, or changes in condition, I promptly report this to the physician.	143 (22.7%)	208 (33.0%)	155 (24.6%)	83 (13.2%)	41 (6.5%)
9. I explain nutritional support, tube protection, and cooperation requirements to patients or their family members.	138 (21.9%)	217 (34.4%)	145 (23.0%)	83 (13.2%)	47 (7.5%)

Data are presented as *n* (%).

### Factors associated with KAP scores

In multivariable linear regression analysis, knowledge scores were significantly lower among female nurses (β = −1.134, 95% CI: −2.214 to −0.054, *p* = 0.040) and nurses without relevant training in the past year (β = −4.676, 95% CI: −5.386 to −3.965, *p* < 0.001), whereas nurses with an intermediate professional title or above had higher knowledge scores (β = 1.200, 95% CI: 0.224 to 2.176, *p* = 0.016). Attitude scores were significantly higher among nurses working in tertiary hospitals (β = 1.049, 95% CI: 0.126 to 1.973, *p* = 0.026), and higher knowledge scores were independently associated with more positive attitudes (β = 0.419, 95% CI: 0.337 to 0.501, *p* < 0.001). Practice scores were significantly lower among nurses without relevant training in the past year (β = −3.014, 95% CI: −4.296 to −1.732, *p* < 0.001), whereas higher knowledge scores (β = 0.860, 95% CI: 0.723 to 0.997, *p* < 0.001) and attitude scores (β = 0.415, 95% CI: 0.294 to 0.537, *p* < 0.001) were independently associated with better practice (Supplementary Tables S3–S5).

### Correlations among KAP scores

Spearman correlation analysis showed that knowledge score was positively correlated with attitude score (*r* = 0.426, *p* < 0.001) and practice score (*r* = 0.616, *p* < 0.001). Attitude score was also positively correlated with practice score (*r* = 0.444, *p* < 0.001) (Supplementary Table S6).

### SEM analysis

The SEM analysis showed acceptable model fit. The χ^2^/df was 3.497, RMR was 0.039, IFI was 0.963, TLI was 0.957, CFI was 0.963, and RMSEA was 0.063, indicating that the hypothesized KAP model adequately fitted the observed data (Supplementary Table S7). In the structural model, knowledge had a significant positive direct effect on both attitude (standardized β = 0.470, 95% CI: 0.404–0.536, *p* < 0.001) and practice (standardized β = 0.512, 95% CI: 0.432–0.589, *p* < 0.001). Attitude had a significant positive direct effect on practice (standardized β = 0.212, 95% CI: 0.135–0.295, *p* < 0.001). In addition, knowledge had an indirect effect on practice through attitude (standardized β = 0.100, 95% CI: 0.061–0.140, *p* < 0.001), with a total effect of 0.611 (95% CI: 0.548–0.672, *p* < 0.001) (Figure 1; Table 5).

**Figure 1:**
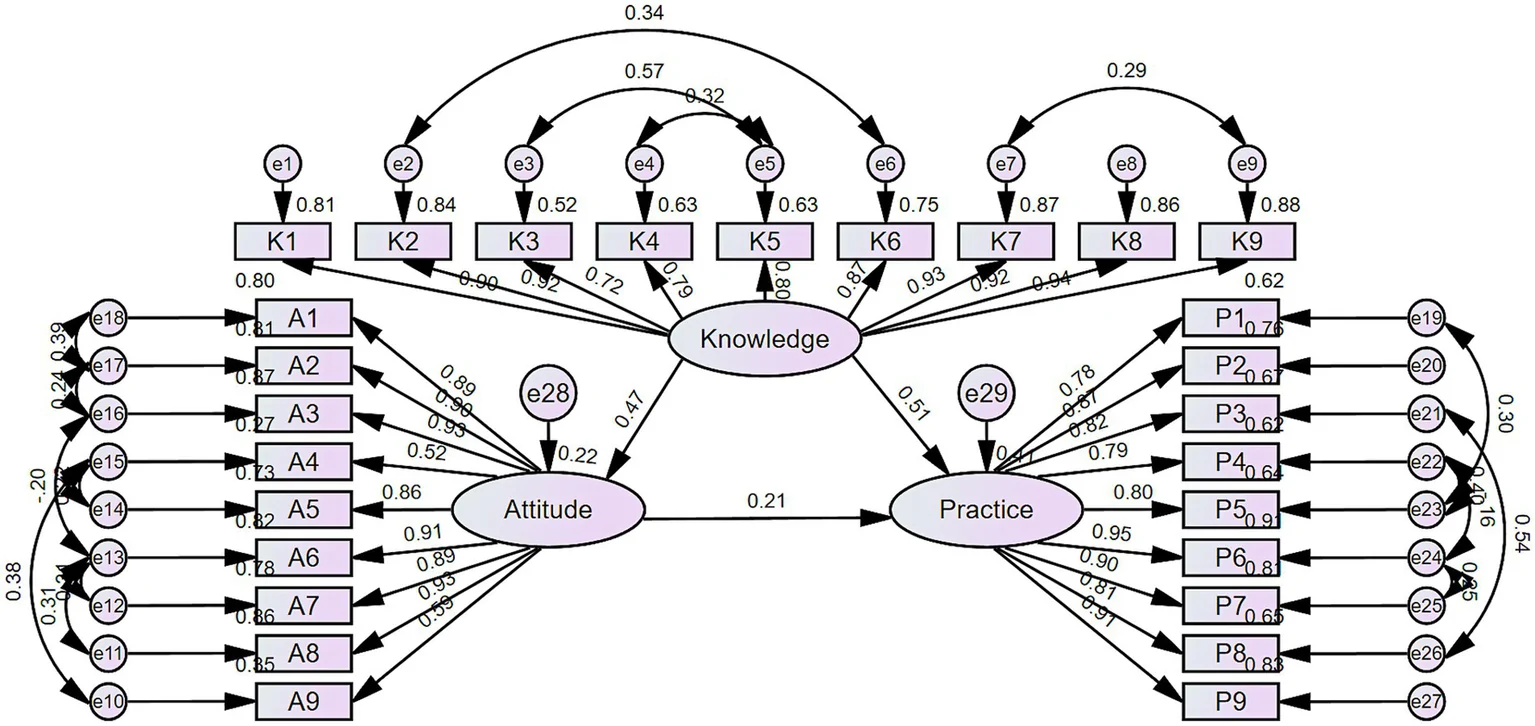
Structural Equation Model (SEM) depicting the relationships among the latent variables: knowledge, attitude, and practice. Ellipses represent latent variables (unobserved constructs), including overall knowledge, attitude, and practice. Rectangles represent observed variables (measured questionnaire items) that serve as indicators of the latent variables. Circles represent error terms or residuals associated with the observed variables. Single-headed arrows indicate hypothesized directional relationships (pathways) between latent variables and from latent variables to observed variables, with standardized path coefficients labeled on the arrows.

**Table 5:** Analysis of direct and indirect effects.

Model paths	Total effects	*p*	Direct effect	*p*	Indirect effect	*p*
Attitude ← Knowledge	0.470 (0.404–0.536)	<0.001	0.470 (0.404–0.536)	<0.001	—	—
Practice ← Knowledge	0.611 (0.548–0.672)	<0.001	0.512 (0.432–0.589)	<0.001	0.100 (0.061–0.140)	<0.001
Practice ← Attitude	0.212 (0.135–0.295)	<0.001	0.212 (0.135–0.295)	<0.001	—	—

β values are standardized estimates. Confidence intervals were calculated using the bias-corrected bootstrap method with 5,000 resamples. CI, confidence interval.

## Discussion

This study showed that ED nurses held insufficient knowledge, generally positive attitudes, and moderate practices regarding nutritional management in patients with sepsis. Training experience in the past year was a significant influence factor associated with knowledge and practice scores. In addition, SEM analyses further supported the theoretical KAP pathway, indicating that knowledge was directly associated with practice and indirectly associated with practice through attitude. These findings highlight the need for structured training and workflow-based support to improve the implementation of nutritional management in emergency sepsis care.

The relatively low knowledge score among nurses warrants particular attention in this study, given that nutritional therapy in sepsis is increasingly recognized as an integral component of comprehensive critical care rather than an optional supportive measure. Current guidelines emphasize nutritional risk assessment, appropriate selection of feeding route, individualized energy and protein provision, and careful monitoring of gastrointestinal tolerance and hemodynamic stability (4, 5, 10). In this study, the weakest knowledge item concerned the use of NRS 2002, NUTRIC, or mNUTRIC for nutritional risk assessment. This finding is noteworthy because NRS 2002 is widely used for hospitalized patients, while NUTRIC and mNUTRIC were specifically developed to identify critically ill patients who may benefit from nutritional intervention (21, 22). Limited familiarity with these tools may delay early recognition of nutritional risk and weaken the continuity of care after ED admission or transfer. Knowledge gaps were also observed in early enteral nutrition, avoidance of early overfeeding, and aspiration risk assessment. The Surviving Sepsis Campaign suggests early enteral nutrition within 72 h in adults with sepsis or septic shock who can be fed enterally (4), while critical care nutrition guidelines caution that feeding should be individualized according to hemodynamic stability, gastrointestinal function, and risk of intolerance (5, 10). The relatively high “do not know” rates for items related to early enteral nutrition and overfeeding indicate that ED nurses may be uncertain about the boundary between beneficial early feeding and unsafe or excessive nutritional delivery. This uncertainty is understandable in emergency settings, where airway, circulation, antimicrobial therapy, and transfer decisions often dominate clinical priorities, but it also highlights a specific educational need.

In contrast to knowledge, nurses’ attitudes were generally positive. More than 90% of participants agreed or strongly agreed that nutritional management is an important component of comprehensive treatment for sepsis and that ED nurses should master relevant knowledge. This indicates that resistance to nutritional care is unlikely to be the main barrier. However, some hesitation remained regarding active identification of feeding contraindications or aspiration risk and inclusion of nutritional management in nursing quality evaluation. This may reflect uncertainty about professional responsibility, concern about additional workload, or lack of clear institutional protocols. Previous studies on sepsis care have similarly shown that nurses’ KAP and clinical decision-making are influenced by training, workload, protocols, interprofessional communication, and organizational support (15, 16). Therefore, attitude alone may be insufficient unless nurses are given clear role definitions and feasible tools within the ED workflow.

The practice dimension revealed more evident implementation gaps. The weakest practices were recording body weight, appetite, and food intake; documenting reasons for enteral nutrition interruption; and assisting with nutritional risk screening. These behaviors are basic but essential for continuity of nutritional care. In critically ill patients, interruptions of enteral nutrition are frequent and are commonly caused by diagnostic or therapeutic procedures, feeding intolerance, tube-related problems, and changes in clinical condition (23, 24). Without systematic documentation, avoidable interruptions may be overlooked, cumulative energy and protein deficits may increase, and subsequent teams may lack information needed for timely adjustment. The finding that about 40% of nurses rarely or never recorded nutrition-related information suggests that the barrier may be embedded in documentation systems and care processes rather than individual motivation alone. Integrating nutrition-related fields into ED nursing records, handover templates, and sepsis care pathways may be more effective than relying solely on personal initiative.

The results of multivariate linear regression indicated that training emerged as the most consistent modifiable factor, especially for knowledge and practice. This is consistent with previous sepsis and nutrition-related KAP studies showing that structured education and protocol-based training are associated with better knowledge, attitudes, and implementation behaviors (15, 25, 26). The SEM findings provide additional support for prioritizing knowledge-oriented educational interventions. Knowledge showed both a substantial direct association with practice and a smaller indirect association through attitude, suggesting that improving nurses’ understanding of nutritional risk assessment, feeding indications and contraindications, aspiration prevention, and interruption management may be relevant not only to attitudes toward nutritional care but also to its practical implementation. This interpretation is also consistent with the multivariable finding that recent training was associated with higher knowledge and practice scores. Nevertheless, because the present study was cross-sectional, these findings should be interpreted as associations rather than evidence that training itself causally improves practice. Prospective educational intervention studies are needed to determine whether targeted training produces sustained improvements in clinical behavior. In addition, nurses with intermediate professional titles or above had higher knowledge scores, possibly because senior nurses have more clinical experience, greater exposure to complex cases, and more opportunities for continuing education (27). Nurses in tertiary hospitals had statistically higher attitude scores. However, the adjusted difference was modest, and its practical significance remains uncertain in the absence of an established minimal clinically important difference for this questionnaire. This association may reflect differences in organizational resources, nutrition support, or institutional protocols, but should be interpreted cautiously. Training remained the more consistent and potentially modifiable factor associated with KAP performance. Training should be practical and scenario-based, covering identified knowledge gaps, such as nutritional screening tools, indications and contraindications for enteral nutrition, aspiration prevention, glucose monitoring, feeding intolerance, and interruption documentation.

The multivariate linear regression, correlation and SEM findings further clarify the mechanism underlying KAP improvement. Knowledge was positively associated with attitude and practice, and attitude was positively associated with practice. SEM showed that knowledge had a strong direct effect on practice and a smaller but significant indirect effect through attitude. This finding is consistent with the KAP theoretical model, which posits that knowledge provides the cognitive basis for attitude formation, whereas attitude facilitates the translation of knowledge into practice and behavior change (28, 29). Importantly, the direct effect of knowledge on practice was stronger than its indirect effect through attitude, suggesting that adequate knowledge may be more immediately linked to nursing practice than attitude change alone. In the context of sepsis nutritional management, knowledge of nutritional risk screening, feeding contraindications, aspiration prevention, and documentation of feeding interruptions may directly affect whether nurses can recognize relevant care needs and implement appropriate practices. Thus, interventions should not be limited to raising awareness, but should also provide practical, guideline-based training that translates key knowledge into standardized bedside actions.

These findings have practical implications for optimizing emergency nursing workflows. Given that the weakest practice items in this study involved nutritional risk screening, recording nutrition-related information, and documenting reasons for enteral nutrition interruption, future quality improvement efforts should focus on embedding these tasks into routine ED sepsis care rather than relying solely on individual initiative. This approach is consistent with current sepsis quality-improvement strategies, which emphasize protocolized screening, structured documentation, multidisciplinary coordination, and integration of sepsis-related activities into ED triage, transitions of care, and broader patient-safety programs (30). In addition, ED nursing workflows may be strengthened by adding concise nutrition-related prompts or checklist items to sepsis nursing records, handover templates, or electronic medical record systems, including nutritional risk screening, feeding status, aspiration risk, and reasons for feeding interruption. Also, 61.3% of nurses in this study had not received relevant training in the past year, and lack of training was independently associated with lower knowledge and practice scores. Training programs should therefore be recurrent, scenario-based, and aligned with standardized protocols, with emphasis on the specific knowledge gaps identified in this study. Finally, if nutrition-related indicators are incorporated into sepsis nursing quality evaluation, role boundaries and workflow feasibility should be clearly defined, since some nurses in this study remained hesitant about including nutritional management in quality assessment. These measures may help translate nurses’ positive attitudes into more consistent, documented, and coordinated nutritional care for patients with sepsis in the ED.

Several limitations should be acknowledged. First, the cross-sectional design precludes causal inference, although SEM provided support for the hypothesized KAP pathway. Second, self-reported practices may be affected by recall bias and social desirability bias; future studies should incorporate chart audits or direct observation. Third, most participants were from public and tertiary hospitals, which may limit generalizability to lower-level or private hospitals. Fourth, the study focused on ED nurses and did not include physicians, dietitians, or ICU nurses, although nutritional management in sepsis is inherently multidisciplinary. Fifth, the convenience sampling strategy and online distribution may have introduced selection bias. In addition, although the questionnaire underwent qualitative expert review for content relevance and clarity, formal quantitative content validity indices were not obtained. Also, the modified Bloom’s cut-offs used to categorize KAP levels are descriptive thresholds commonly adopted in KAP research rather than clinically validated standards. Therefore, these categorical interpretations should be considered exploratory. Because individual hospital identifiers and recruitment-source identifiers were not collected, the number of respondents contributed by each hospital could not be reconstructed, and potential within-hospital clustering could not be quantified using an intraclass correlation coefficient or incorporated into cluster-adjusted analyses. Consequently, the effective independent sample size may be smaller than the nominal sample size, and the precision of the reported associations may be somewhat overestimated. Training experience was assessed as a binary self-reported variable. Detailed characteristics, including training content, format, duration, provider, mandatory status, and competency assessment, were not collected. Therefore, the observed association reflects exposure to recent nutrition-related training in general and cannot determine which training characteristics are most effective. Finally, intervention studies are needed to determine whether targeted training and workflow optimization can improve nurses’ practices and patient-level nutritional outcomes. Future studies should use prospective, multicenter designs with more representative sampling, explicit hospital-level identifiers, and comprehensive psychometric validation of the questionnaire and score thresholds. More detailed assessment of training characteristics, together with objective practice measures and patient-level nutritional outcomes, is also needed to determine whether targeted education and workflow interventions can produce meaningful and sustained improvements in sepsis nutritional care.

In conclusion, ED nurses showed insufficient knowledge, positive attitudes, and moderate practices regarding nutritional management in patients with sepsis. Training, professional title, knowledge, and attitude were associated with KAP performance. The observed direct and indirect associations of knowledge with practice, together with the association between recent training and KAP performance, support the potential value of targeted, practice-oriented education. Standardized documentation and workflow-based support may further facilitate translation of knowledge into consistent nutritional care.

## Data Availability

The raw data supporting the conclusions of this article will be made available by the authors, without undue reservation.

## Footnotes

1
https://www.wjx.cn/
