# Supplementary material for: Knowledge, attitudes, and practices of emergency nurses regarding sepsis nutritional management: a multicenter cross-sectional study
Source: Front Nutr. 2026 Oct 8;13:1933045. doi: 10.3389/fnut.2026.1933045 (PMC13687387; doi:10.3389/fnut.2026.1933045)
Supplement: Supplementary file 1 [file Table_1.docx]

**Table S1. Confirmatory factor analysis model-fit indices**

| **Fit index** | **Recommended threshold** | **Result** |
| --- | --- | --- |
| CMIN/DF | <3, excellent; <5, good | 3.497 |
| RMR | <0.080 | 0.039 |
| IFI | >0.900 | 0.963 |
| TLI | >0.900 | 0.957 |
| CFI | >0.900 | 0.963 |
| RMSEA | <0.080 | 0.063 |

**Note:** CMIN/DF, chi-square/degrees of freedom ratio; RMR, root mean square residual; IFI, incremental fit index; TLI, Tucker–Lewis index; CFI, comparative fit index; RMSEA, root mean square error of approximation.

**Table S2. Standardized factor loadings of the confirmatory factor analysis**

| **Path** | **Estimate** | **SE** | **CR** | **P** | **Standardized β** | **95% BC CI for β** |
| --- | --- | --- | --- | --- | --- | --- |
| Knowledge → K1 | 1 | — | — | — | 0.902 | 0.867–0.928 |
| Knowledge → K2 | 1.032 | 0.027 | 37.849 | <0.001 | 0.916 | 0.891–0.938 |
| Knowledge → K3 | 0.806 | 0.035 | 22.875 | <0.001 | 0.721 | 0.669–0.773 |
| Knowledge → K4 | 0.915 | 0.034 | 27.156 | <0.001 | 0.793 | 0.745–0.833 |
| Knowledge → K5 | 0.875 | 0.032 | 27.273 | <0.001 | 0.796 | 0.754–0.833 |
| Knowledge → K6 | 0.978 | 0.03 | 32.916 | <0.001 | 0.868 | 0.832–0.899 |
| Knowledge → K7 | 1.088 | 0.028 | 39.526 | <0.001 | 0.931 | 0.903–0.951 |
| Knowledge → K8 | 1.057 | 0.027 | 38.615 | <0.001 | 0.925 | 0.900–0.945 |
| Knowledge → K9 | 1.089 | 0.027 | 40.312 | <0.001 | 0.937 | 0.916–0.954 |
| Attitude → A9 | 1 | — | — | — | 0.588 | 0.525–0.649 |
| Attitude → A8 | 1.08 | 0.063 | 17.123 | <0.001 | 0.927 | 0.904–0.945 |
| Attitude → A7 | 1.005 | 0.06 | 16.708 | <0.001 | 0.886 | 0.850–0.916 |
| Attitude → A6 | 1.034 | 0.061 | 16.824 | <0.001 | 0.905 | 0.875–0.930 |
| Attitude → A5 | 1.045 | 0.064 | 16.396 | <0.001 | 0.855 | 0.809–0.893 |
| Attitude → A4 | 0.983 | 0.069 | 14.336 | <0.001 | 0.515 | 0.448–0.583 |
| Attitude → A3 | 1.038 | 0.061 | 17.132 | <0.001 | 0.933 | 0.908–0.953 |
| Attitude → A2 | 0.998 | 0.06 | 16.772 | <0.001 | 0.898 | 0.865–0.926 |
| Attitude → A1 | 0.963 | 0.058 | 16.712 | <0.001 | 0.892 | 0.854–0.926 |
| Practice → P1 | 1 | — | — | — | 0.784 | 0.743–0.821 |
| Practice → P2 | 1.076 | 0.043 | 25.292 | <0.001 | 0.87 | 0.837–0.896 |
| Practice → P3 | 0.979 | 0.042 | 23.281 | <0.001 | 0.821 | 0.779–0.857 |
| Practice → P4 | 0.991 | 0.045 | 22.045 | <0.001 | 0.787 | 0.738–0.828 |
| Practice → P5 | 0.943 | 0.035 | 27.037 | <0.001 | 0.802 | 0.757–0.838 |
| Practice → P6 | 1.136 | 0.04 | 28.478 | <0.001 | 0.953 | 0.936–0.966 |
| Practice → P7 | 1.068 | 0.041 | 26.168 | <0.001 | 0.901 | 0.868–0.926 |
| Practice → P8 | 0.951 | 0.042 | 22.79 | <0.001 | 0.807 | 0.765–0.846 |
| Practice → P9 | 1.09 | 0.041 | 26.721 | <0.001 | 0.911 | 0.886–0.932 |

Note: Standardized β represents the standardized factor loading. BC CI, bias-corrected bootstrap confidence interval. Bootstrap confidence intervals were calculated using 5,000 resamples.

**Table S3. Factors associated with knowledge scores**

| **Variable** | **Univariable linear regression analysis β (95% CI)** | **P** | **Multivariable linear regression analysis β (95% CI)** | **P** |
| --- | --- | --- | --- | --- |
| **Sex** |  |  |  |  |
| Male | Reference | — | Reference | — |
| Female | -1.290 (-2.519 to -0.063) | 0.039 | -1.134 (-2.214 to -0.054) | 0.040 |
| **Age, years** |  |  |  |  |
| ≤34 | Reference | — | Reference | — |
| 35–39 | 1.350 (0.322 to 2.378) | 0.01 | 1.058 (-0.156 to 2.273) | 0.088 |
| ≥40 | 2.050 (1.174 to 2.923) | <0.001 | 0.842 (-0.628 to 2.313) | 0.261 |
| **Educational level** |  |  |  |  |
| Junior college or below | Reference | — | — | — |
| Bachelor’s degree or above | 0.661 (-0.176 to 1.498) | 0.122 | — | — |
| **Work experience, years** |  |  |  |  |
| ≤5 | Reference | — | Reference | — |
| 6–10 | 1.040 (-0.215 to 2.292) | 0.104 | 0.303 (-0.845 to 1.451) | 0.605 |
| 11–20 | 1.990 (0.827 to 3.158) | <0.001 | -0.172 (-1.672 to 1.329) | 0.823 |
| ≥21 | 2.910 (1.634 to 4.186) | <0.001 | 0.159 (-1.710 to 2.029) | 0.867 |
| **Professional title** |  |  |  |  |
| Junior title or below | Reference | — | Reference | — |
| Intermediate title or above | 2.120 (1.253 to 2.981) | <0.001 | 1.200 (0.224 to 2.176) | 0.016 |
| **Hospital type** |  |  |  |  |
| Public hospital | Reference | — | — | — |
| Private hospital | 1.360 (-0.683 to 3.407) | 0.191 | — | — |
| **Hospital level** |  |  |  |  |
| Non-tertiary hospital | Reference | — | — | — |
| Tertiary hospital | 0.380 (-0.609 to 1.368) | 0.451 | — | — |
| **Training experience in the past year** |  |  |  |  |
| Yes | Reference | — | Reference | — |
| No | -4.870 (-5.578 to -4.162) | <0.001 | -4.676 (-5.386 to -3.965) | <0.001 |

Note: β, regression coefficient; CI, confidence interval. Reference indicates the reference category in the regression model. Variables not included in the multivariable model are indicated by “—”.

**Table S4. Factors associated with Attitude scores**

| **Variable** | **Univariable linear regression analysis β (95% CI)** | **P** | **Multivariable linear regression analysis β (95% CI)** | **P** |
| --- | --- | --- | --- | --- |
| **Sex** |  |  |  |  |
| Male | Reference | — | Reference | — |
| Female | 0.239 (-0.996 to 1.473) | 0.704 | 0.772 (-0.361 to 1.905) | 0.182 |
| **Age, years** |  |  |  |  |
| ≤34 | Reference | — | Reference | — |
| 35–39 | 0.754 (-0.287 to 1.794) | 0.155 | -0.270 (-1.541 to 1.001) | 0.677 |
| ≥40 | 1.350 (0.469 to 2.240) | 0.003 | -0.224 (-1.762 to 1.314) | 0.775 |
| **Educational level** |  |  |  |  |
| Junior college or below | Reference | — | — | — |
| Bachelor’s degree or above | 0.122 (-0.719 to 0.962) | 0.776 | — | — |
| **Work experience, years** |  |  |  |  |
| ≤5 | Reference | — | Reference | — |
| 6–10 | 0.659 (-0.608 to 1.926) | 0.308 | 0.200 (-0.999 to 1.399) | 0.744 |
| 11–20 | 1.460 (0.278 to 2.633) | 0.015 | 0.763 (-0.804 to 2.329) | 0.34 |
| ≥21 | 2.140 (0.848 to 3.427) | 0.001 | 1.231 (-0.723 to 3.184) | 0.217 |
| **Professional title** |  |  |  |  |
| Junior title or below | Reference | — | Reference | — |
| Intermediate title or above | 1.260 (0.381 to 2.134) | 0.005 | 0.081 (-0.944 to 1.106) | 0.877 |
| **Hospital type** |  |  |  |  |
| Public hospital | Reference | — | — | — |
| Private hospital | -0.321 (-2.373 to 1.731) | 0.759 | — | — |
| **Hospital level** |  |  |  |  |
| Non-tertiary hospital | Reference | — | Reference | — |
| Tertiary hospital | 1.000 (0.014 to 1.990) | 0.047 | 1.049 (0.126 to 1.973) | 0.026 |
| **Training experience in the past year** |  |  |  |  |
| Yes | Reference | — | Reference | — |
| No | -2.150 (-2.943 to -1.365) | <0.001 | 0.053 (-0.784 to 0.890) | 0.901 |
| **Knowledge score** | 0.429 (0.358 to 0.500) | <0.001 | 0.419 (0.337 to 0.501) | <0.001 |

Note: β, regression coefficient; CI, confidence interval. Reference indicates the reference category in the regression model. Variables not included in the multivariable model are indicated by “—”.

**Table S5. Factors associated with Practice scores**

| **Variable** | **Univariable linear regression analysis β (95% CI)** | **P** | **Multivariable linear regression analysis β (95% CI)** | **P** |
| --- | --- | --- | --- | --- |
| **Sex** |  |  |  |  |
| Male | Reference | — | Reference | — |
| Female | -0.487 (-2.807 to 1.832) | 0.680 | 0.567 (-1.170 to 2.303) | 0.522 |
| **Age, years** |  |  |  |  |
| ≤34 | Reference | — | Reference | — |
| 35–39 | 0.895 (-1.065 to 2.855) | 0.37 | -0.895 (-2.376 to 0.586) | 0.236 |
| ≥40 | 2.080 (0.417 to 3.752) | 0.014 | -1.350 (-2.883 to 0.184) | 0.085 |
| **Educational level** |  |  |  |  |
| Junior college or below | Reference | — | — | — |
| Bachelor’s degree or above | 0.260 (-1.320 to 1.839) | 0.747 | — | — |
| **Work experience, years** |  |  |  |  |
| ≤5 | Reference | — | — | — |
| 6–10 | 1.500 (-0.900 to 3.890) | 0.221 | — | — |
| 11–20 | 1.580 (-0.647 to 3.805) | 0.164 | — | — |
| ≥21 | 2.910 (0.476 to 5.351) | 0.019 | — | — |
| **Professional title** |  |  |  |  |
| Junior title or below | Reference | — | Reference | — |
| Intermediate title or above | 3.220 (1.581 to 4.856) | <0.001 | 1.270 (-0.271 to 2.810) | 0.107 |
| **Hospital type** |  |  |  |  |
| Public hospital | Reference | — | — | — |
| Private hospital | 2.670 (-1.178 to 6.523) | 0.173 | — | — |
| **Hospital level** |  |  |  |  |
| Non-tertiary hospital | Reference | — | — | — |
| Tertiary hospital | 0.491 (-1.371 to 2.353) | 0.605 | — | — |
| **Training experience in the past year** |  |  |  |  |
| Yes | Reference | — | Reference | — |
| No | -8.070 (-9.448 to -6.694) | <0.001 | -3.014 (-4.296 to -1.732) | <0.001 |
| **Knowledge score** | 1.170 (1.058 to 1.289) | <0.001 | 0.860 (0.723 to 0.997) | <0.001 |
| **Attitude score** | 0.842 (0.711 to 0.974) | <0.001 | 0.415 (0.294 to 0.537) | <0.001 |

Note: β, regression coefficient; CI, confidence interval. Reference indicates the reference category in the regression model. Variables not included in the multivariable model are indicated by “—”.

**Table S6. Correlation analysis**

| **Spearman** | **Knowledge** | **Attitude** | **Practice** |
| --- | --- | --- | --- |
| **Knowledge** | 1.000 |  |  |
| **Attitude** | 0.426 (P<0.001) | 1.000 |  |
| **Practice** | 0.616 (P<0.001) | 0.444 (P<0.001) | 1.000 |

**Table S7. SEM fit indicators**

| **Fit index** | **Recommended threshold** | **Result** |
| --- | --- | --- |
| CMIN/DF | <3, excellent; <5, good | 3.497 |
| RMR | <0.080 | 0.039 |
| IFI | >0.900 | 0.963 |
| TLI | >0.900 | 0.957 |
| CFI | >0.900 | 0.963 |
| RMSEA | <0.080 | 0.063 |

**Note:** SEM, structural equation modeling; CMIN/DF, chi-square/degrees of freedom ratio; RMR, root mean square residual; IFI, incremental fit index; TLI, Tucker–Lewis index; CFI, comparative fit index; RMSEA, root mean square error of approximation.
